# Polish Pharmacy Students’ Attitudes toward Undergraduate Teaching and Practical Implementation of Pharmaceutical Care—A Cross Sectional Study

**DOI:** 10.3390/ijerph19127358

**Published:** 2022-06-15

**Authors:** Beata Plewka, Magdalena Waszyk-Nowaczyk, Magdalena Cerbin-Koczorowska, Michał Michalak, Aleksandra Sajko, Monika Bańdurska, Tomasz Osmałek

**Affiliations:** 1 Departmentof Pharmaceutical Technology, Pharmacy Practice Division, Poznan University of Medical Sciences, 60-780 Poznan, Poland; mwaszyk@ump.edu.pl; 2 Department of Medical Education, Poznan University of Medical Sciences, 61-701 Poznan, Poland; mcerbin@ump.edu.pl; 3 Department of Computer Science and Statistics, Poznan University of Medical Sciences, Rokietnicka 7 St., 61-806 Poznan, Poland; michal@ump.edu.pl; 4 Student’s Pharmaceutical Care Group, Department of Pharmaceutical Technology, Pharmacy Practice Division, Poznan University of Medical Sciences, 60-780 Poznan, Poland; aleksandra.sajko1@gmail.com (A.S.); monikabandurska97@gmail.com (M.B.); 5 Department of Pharmaceutical Technology, Poznan University of Medical Sciences, Grunwaldzka 6, 60-780 Poznan, Poland; tosmalek@ump.edu.pl

**Keywords:** pharmaceutical care, pharmacy students, pharmacy curriculum

## Abstract

It is necessary to monitor and adapt pharmacy curricula to make graduates ready to effectively meet the health needs of the society. Therefore, the aim of the study was to obtain Polish final year pharmacy students’ opinions on the activities related to pharmaceutical care (PC) andtheir perception of PC. Two questionnaires were used: one evaluating a PC regarding classes and in practice (*n* = 64), and the other on the assessment of existing educational solutions in the field of professional counseling (*n* = 118). Statistical analysis showed that the students agreed that there are not enough hours of a PC classes, since these are necessary to enable them to provide PC services in the future (R = 0.05, *p* = 0.0007). In previous classes, teachers’ knowledge was rated the highest on a five-point scale (4.74), and role play (3.92) and the duration of the classes (3.77) were rated the lowest. Although the students were aware of the role of a PC in the work of a pharmacist, they indicated that more extensive use of active learning methods would better prepare them for the profession. Therefore, pharmacy students’ self-efficacy survey and a curriculum renewal would be recommended in Poland.

## 1. Introduction

The International Pharmaceutical Federation defined the competences of pharmacists in community pharmacies as the provision of four basic services: prescribing drugs, pharmacotherapy verification, prescription fulfillment, and drug administration (including vaccinations) [1]. Each of these services is a form of pharmaceutical care (PC), which, according to the definition adopted by the Polish legislature in the Act of 10 December 2020 on the profession of pharmacist, based on the definition of Hepler and Strand, 1990, is “a documented process in which a pharmacist, working with the patient and the physician treating the patient, and, if necessary, with other medical professions, watches over the proper course of individual pharmacotherapy ” [2,3]. The introduction of PC into pharmacy practice has become the main goal of pharmacists in many countries in Europe and around the world [4,5]. In Poland, before the aforementioned act on the profession of pharmacist, this was reflected in the Regulation of the Minister of Science and Higher Education of 26 July 2019, which presented the standards of pharmacy undergraduate curriculum, including the learning outcomes in the field of PC (Table 1) [6]. They are in line with the standards adopted by the European Association of Faculties of Pharmacy, which define the four pillars of pharmaceutical education: science-practice balance, experiential teaching methods, interdisciplinary methods, and preparedness for lifelong learning [7]. In the United States, the Accreditation Council for Pharmacy Education (ACPE) plays a similar role, which, among other things, offers a program of certification of pharmaceutical courses on the assumption that their graduates will be prepared to provide patient care in order to optimize the safety and effectiveness of medicine use [8].

Although it includes most of the above-mentioned aspects of running PC, the research conducted so far suggests that graduates do not always feel prepared to engage in activities that go beyond professional tasks related to trade in medicinal products [9]. Additionally, the COVID-19 pandemic has emphasized the role of pharmacists as the frontline health professionals to whom patients have the easiest access. This, in turn, resulted in an increase in their competences in the Polish health system [10]. Since 1 April 2020, pharmacists in Poland have been allowed issue pharmaceutical prescriptions for patients and prescriptions for themselves and their family (pro auctore/pro familia) for all medicines with category “Rp,” prescribed by a physician, with the exception of drugs containing substances listed in the Act on counteracting drug addiction, e.g., drugs or psychotropics. Moreover, they were enabled to undergo courses authorizing them to vaccinate against COVID-19. The recruitment of pharmacies wishing to join the National Immunization Program began in June 2021. Although all these changes, partially accelerated by the pandemic situation, were necessary for the implementation of the idea of pharmaceutical care in Poland, many pharmacy employees still do not feel prepared to perform new tasks. Their attitudes towards involvement in pharmaceutical services are also varied. The lack of time and the undefined role of the pharmacist in the healthcare system, including the lack of a clear division of competences between pharmacists and physicians were highlighted among the significant barriers [9,11,12].

In view of such a dynamically changing role of the pharmacist in the health care system, it is necessary to constantly monitor and adapt curriculum to enable the education of graduates who are ready to effectively meet the health needs of the society. Pursuant to the Act on the Pharmacist’s Profession, the new tasks of a pharmacist in a community pharmacy will include PC services, such as medicines use review, developing an individual PC plan, and performing diagnostic tests [2]. The process by which the curriculum is revised and refreshed is called curriculum renewal, and one of its basic elements is to know the expectations of students and then to evaluate the changes made [13,14]. According to the assumptions of the outcome-based education approach, the first step in initiating changes should be the precise definition of learning outcomes, the achievement of which will allow the graduate to correctly implement the entrusted professional tasks. Based on these assumptions, decisions will be made in the field of educational content, taking into account the current state of knowledge and didactic methods enabling the acquisition of qualifications, not only in the knowledge domain, but also in the area of social skills and competences.

According to the assumptions of the Kirkpatrick model, one of the most frequently used tools in the evaluation of educational activities, the first and basic level of evaluation of training effectiveness should be the measurement of the level of satisfaction and commitment of its participants and their belief that the acquired knowledge will be useful in their later professional work [15]. Although there is no doubt that pharmacy students in Poland acquire extensive knowledge during their studies, they have too few opportunities to learn how to use this knowledge in practice while working with patients, taking into account the specificity of professional tasks faced by graduates. That is why we decided to get to know the opinions and expectations of fifth year pharmacy students regarding the curriculum, as well as the very idea of PC as a service provided in pharmacies. In addition to the educational content, we also took into account the aspects that make up the proper organization of the educational environment. Research shows that elements such as the atmosphere, the number of learning opportunities, and the educational equipment are important for student involvement, their satisfaction with the course, and the achievement of the intended learning outcomes. For instance, students mention having a stressless learning environment, proper equipment, such as adequate internet speed, up to date scientific resources, or ergonomic chairs, and opportunities for the practical application of knowledge as a relevant factors affecting their learning [16,17].

The research is part of the curriculum renewal project and consisted of two questionnaires, one used in first stage and one in the second stage, the results of which will allow for changes in line with the students’ expectations. In the first stage, an assessment was made of the perception of the principles of PC implementation in future professional work. Students’ expectations in the context of educational content and methods that should be included in teaching PC were also examined. Knowing the expectations of students will allow for the future design of classes that best suit their needs. In the second stage, the currently delivered educational solutions were evaluated based on the assumptions of level 1 of the Kirkpatrick model. This will help to identify the weakest and strongest points of the current curriculum. The assessed course was conducted at the Pharmacy Practice Division in the 9th semester of the pharmacy program and concerned the issue of practical pharmacy in a community pharmacy. Students participated in three modules, during which aspects of professional counseling in respiratory, digestive, and venous diseases were discussed, using presentations, role plays, and case studies. Pharmacy studies in Poland last 11 semesters, the last of which is a six-month apprenticeship in a community and hospital pharmacy. Before that, students complete two summer internships: one month after the third year at a community pharmacy, and another month after the fourth year at a hospital pharmacy, with the option of dividing their time among pharmacy and another place, e.g., a pharmaceutical company or a laboratory. Graduates obtain a master’s degree after the tenth semester, while completing a six-month internship within the eleventh semester allows them to obtain the license to practice as a pharmacist. All students are currently following the same study program, regardless of the chosen path of the future professional career. They choose a specific place of work after graduation: community pharmacy, hospital pharmacy, pharmaceutical industry, or laboratory.

## 2. Materials and Methods

A cross-sectional questionnaire-based study was performed. The study plan consisted of two phases in which two different questionnaires were used. The first concerned the students’ opinion on a PC as a general ideadiscussed during studies and PC in community pharmacy practice. The second questionnaire was used to examine students’ opinions on professional counseling classes they attended during the 9th semester. The study was conducted among 139 students of the fifth year of pharmacy in the 2019/2020 academic year at the Poznan University of Medical Sciences (PUMS) who completed most of the courses provided for in the study program, and who were close to starting their professional careers. The participation in the study was anonymous and voluntary. Prior the study, all participants were informed about its purpose and the freedom to withdraw from participation at each of its stages. They were invited to ask questions regarding the study, and any concerns were resolved. The surveys were conducted from October 2019 to February 2020. The study protocol was reviewed by the Bioethics Committee at the Poznan University of Medical Sciences on 16 January 2020. All collected data were securely stored in the Department of Pharmaceutical Technology, Pharmacy Practice Division, at PUMS.

First, questionnaire was divided into two parts. The first part contained 18 statements about the teaching of PC during studies, and in the second, there were 15 statements that concerned conducting PC in practice. The questionnaire was subject to content validity assessment, with the participation of four experts, two in the field of medical education, one academic teacher conducting classes on pharmaceutical counseling and care, and one pharmacist practicing in a community pharmacy. Students were invited to complete a questionnaire after completing the Pharmaceutical Care module for three chronic diseases: asthma, diabetes, and hypertension. The demographic structure of students is presented in Table 2. Eighty-three questionnaires were distributed, 64 of which were returned, and all of them were included in the study (response rate at 77.1%). Although the total number of students was 139, not every teacher issued the questionnaires, due to time constraints. Surveyed students marked used a five-point Likert scale to state the extent to which a given statement was consistent with their beliefs, where 1 was for “strongly disagree” and 5 for “strongly agree.” The obtained results were analyzed statistically. The variables were compared via determination of the Spearman’s rank correlation coefficient. The results were considered statistically significant with *p* < 0.05. The thematic scope of the survey is presented in Table 3 and Table 4.

In the second questionnaire, the currently delivered educational solutions were evaluated based on the assumptions of level 1 of the Kirkpatrick model. The surveys were conducted among fifth-year pharmacy students who participated in classes on professional counseling at the community pharmacy in the field of selected diseases. The demographic structure of respondents is presented in Table 5. The students were asked by the tutors at the end of the last class in the module to fill out the questionnaires. Students who consented to participate in the study were allowed to remain in the class to complete the survey. Everyone took advantage of this solution. A total of 139 forms were issued and 118 questionnaires were returned and qualified for the study (response rate at 84.9%). Students assessed the classes in terms of eight aspects, using a five-point rating scale, where 1 meant the lowest rating (“very bad”) and 5 the highest (“very good”). The questionnaire also included questions about the knowledge of the learning outcomes adopted for professional counseling classes, as well as preferred forms and methods of teaching. The results were analyzed statistically. For the comparison of two independent groups, when the variable was measured on an ordinal scale, the Mann–Whitney U test (with continuity correction) was used. In turn, the Friedman test was used to compare the differences between at least three dependent groups, when the variable was measured on an ordinal scale. The results were considered statistically significant with *p*<0.05.

## 3. Results

### 3.1. Pharmaceutical Care Survey

Students identified practical aspects as the most important issues discussed during the PC classes, i.e., the use of self-monitoring devices (e.g., glucometers or blood pressure monitors) and specific drug dosage forms (e.g., inhalers), as well as conducting an interview with the patient, and detecting drug problems and interactions. By comparing the answers in the first part of the questionnaire (Table 3) with the answers in the second part (Table 4), it was possible to identify the relationship between the perception of the PC as an element of the curriculum and the attitude towards the provision of PC services in a community pharmacy. It turned out that the students strongly agreed with the statement that the curriculum did not provided enough hours of classes related to PC, which is a consequence of the belief that they should be able to provide services in the field of PC in the future (R = 0.4, *p* = 0.0007). At the same time, the respondents recognized that PC classes can positively affect their preparation for professional work, and the provision of services in the field of a PC does not encroach on physicians’ competencies. The future pharmacists not only agreed that the classes related to PC should be one of the basic curriculum elements, which should be included in the compulsory classes earlier than in the 5th year of studies, but also that the service itself is one of the basic professional duties of pharmacists working in community pharmacies (R = 0.5, *p* = 0.0120). Moreover, it was pointed out that a PC classes should cover practical aspects, such as the use of self-control devices or various drug forms (e.g., inhalers), but with the belief that a PC is not limited to people with chronic diseases.

One of the aspects covered in the survey included previous experiences in their studies that affected students’ self-confidence related to running a PC. The students pointed out that the previous classes prepared them well in terms of knowledge of the use of self-control devices, and in this aspect, running a PC has a real impact on patients’ health. The possibility of learning to run a PC during apprenticeships also turned out to be an important issue, because in the opinion of students, a PC is the responsibility of pharmacists (*p* = 0.0001) and has a real impact on patients’ health (R = 0.4, *p* = 0.0002). Moreover, as the conviction of having appropriate competences in the field of PC grows, the students’ conviction that a PC is a pharmacist’s duty grows, and the conviction that PC should be provided only by specialized pharmacists decreases (R = 0.4, *p* = 0.0025). It is also worth mentioning that the group of students who agree with the statement that PC should be mainly taught during elective courses and that the number of a PC classes in the currentcurriculum is sufficient, is also strongly linked to the people with opposite attitudes, that the PC is not a pharmacist’s obligation and is part of the physicians’, not pharmacists’, competences. Moreover, they believe that pharmacists should not be required to run the PC.

### 3.2. Evaluation Survey

As part of the survey evaluating the classes, students assessed individual aspects in three thematic modules in the field of professional consulting in diseases of the digestive (module X), respiratory (module Y), and venous systems (module Z). Within the individual modules, the following number of questionnaires was obtained: X—112, Y—109, Z—115. As no statistically significant differences were obtained in the assessment of individual modules, the results were averaged for further consideration. The grading scale was adopted in the range of 1–5, where 1 was for “very bad” and 5 for “very good”. Students rated the activities in seven categories: overall rating, location and duration of the classes, the lecturer’s knowledge and multimedia presentation, as well as cases and role-playing scenes used by the lecturers. Students were also allowed to enter their own comments regarding individual modules. As a result, the respondents rated the classes at 4.38 (*n* = 338), and the highest rated aspects were the knowledge of the teachers (4.74; *n* = 340) and the cases considered during the classes (4.31; *n* = 340). The multimedia presentations and the locations of the classes were also well rated (4.18; *n* = 340, and 4.01; *n* = 341, respectively), while the lowest scores were given to the proposed role-playing scenes (3.92; *n* = 331) and the duration of classes (3.77; *n* = 341). Comments posted by the respondents indicated that the lowest score for the use of role-play was most often given by students who participated in the classes in which the tutor did not use this method at all. Nineteen people indicated that the scenes were not used, or there were not enough of them. Three people indicated that the scenario of the role play was unrealistic. Although the general assessment of the knowledge of the teachers was high, it should be noted that the comments often mentioned that too much theory was presented during the classes (14 replies), the teacher spoke too quickly (5 replies), and the teacher did not activate the students (2 replies), which translated into the feeling that the classes involve too few practical PC aspects (10 replies).The students also assessed the extent to which the activities they participated in made them feel good about the use of knowledge in practice, and in this aspect, their average mark was 4.13.

However, as many as 66.1% (*n* = 78) of students admitted that they did not know the learning outcomes adopted for the professional counseling course. Twenty-two students (18.6%) decided that they knew the effects and listed them. Eighteen students did not answer that question (15.3%). In general terms, the learning outcomes mentioned by students correspond to the outcomes envisaged in the syllabus for the subject. However, it is noteworthy that the syllabus foresees 6 specific effects for knowledge, 10 for skills, and 4 for competences. Among these are learning outcomes, such as knowledge of the tools and principles of PC documentation, or the ability to prepare a PC or patient education plan meeting the standards of the legal bases of running a PC. None of these was specifically defined by students. Instead, when asked to name the learning outcomes, they described them as acquisition of professional advisory skills, the ability to select medicines for the patient, communication/contact with the patient, acquiring practical skills, better patient care, preparation for work in a pharmacy, and practical application of knowledge from studies or self-study of cases. It is worth emphasizing that although the study concerned only PUMS students, the learning outcomes in Poland are standardized and included in the aforementioned Regulation of the Minister of Science and Higher Education. The universities are free to choose the content and educational methods.

## 4. Discussion

### 4.1. Pharmacy Students Career Paths

The vast majority of graduates of pharmaceutical faculties in Poland begin their professional careers in community pharmacies. For instance, at Poznan University of Medical Sciences, statistics conducted by the Promotion and Career Department show that graduates who completed their studies in the 2018/2019 and 2019/2020 academic years often started their first job in a community pharmacy–83.3% and 46.4%, respectively. However, among the aspects that, in their opinion, should be included in the curriculum in order to better prepare them for their professional work, they mentioned primarily the need for more hours of practical activities regarding pharmaceutical care and communication with patients. According to similar reports presented annually by the career office of the Jagiellonian University of Krakow, 42.9% of 2018/2019 graduates chose a pharmacy as their workplace, and 9.5% chose a hospital (without specifically mentioning a hospital pharmacy as their workplace). The data for graduates from 2019/2020 are comparable, although a larger number of people from this academic year indicated the hospital—34.8% vs. 21.7%, respectively. In both reports, the respondents indicated that after graduation, they lacked practical skills (e.g., in the field of operating pharmacy programs) and preparation for direct work with patients [18,19]. The cited reports touch upon two important aspects that were highlighted in the surveys we conducted. One is related to the choice of a pharmacy graduates’ career path, and thus, specific professional tasks, and the other is related to the preparation of students for their implementation.

### 4.2. Pharmaceutical Care as an Apothecary’s Task

Graduates who choose a community pharmacy as their place of work are obliged by the act on the profession of pharmacist to provide pharmaceutical care and pharmaceutical services [2]. In the survey on pharmaceutical care, the majority of students expressed the opinion that pharmaceutical care is an obligatory task of a community pharmacist, and therefore, classes related to this subject should be also obligatory and present from the first years of study. However, there was a group of 20 students who expressed the opposite opinion. According to them, running a PC is not only not the responsibility of pharmacists, but it is part of the competences of physicians, which was related to the belief that there are enough hours of classes regarding a PC during their studies. Taking into account the statistics on the choice of employment by pharmacy graduates, they could be people who do not associate their professional career with a community pharmacy. On the other hand, the research conducted among students of pharmacy at the Medical University of Lublin led the authors to the conclusion that students who declare that their professional career is associated with a community pharmacy exhibit more conservative attitudes towards the PC, which may result in greater involvement in tasks related directly to dispensing drugs than with running a PC [20]. The solution seems to be greater specialization during studies and the creation of detailed descriptions of career paths to help students choose the most appropriate profile [21].

### 4.3. Teaching Pharmaceutical Care

In our PC survey, it was pointed out that there are too few PC classes, because those in which the students have participated so far have prepared them well for the practical implementation of the discussed issues (e.g., handling tests for self-examination), but have not exhausted all aspects of a PC, according to the definition provided in in the Act of 10 December 2020 on the profession of pharmacist. Thus, students feel that every opportunity to gain practical knowledge during their studies allows them to better respond to the health needs of patients in the future. Similar results were obtained in the studies by Raczkiewicz etal., also carried out in 2019, among students of the fourth year of pharmacy training. The authors of the study distinguished aspects of readiness to promote health in pharmacies at the level of pharmacy students, pharmacy employees, and individual self-belief. The results showed that students negatively assessed the readiness of pharmacy students and pharmacists to provide such a service, although individually, they believed that they were ready to promote health in their future work [22]. Most importantly, despite the indicated limitations in systemic solutions that hinder the promotion of health in pharmacies, in term of running a PC, students in our study showed high motivation to provide such a service. Moreover, in the study conducted in Poland by Świeczkowski et al., pharmacy students, as well as medical students, expressed a positive attitude towards running a PC in community pharmacies [23]. Additionally, future pharmacists are ready to establish cooperation with representatives of other medical professions in order to optimize the patient’s therapy, which was confirmed by the research conducted by Cerbin-Koczorowska et al. [24]. On the other hand, research conducted in Qatar and Kuwait among final-year pharmacy students showed that students feel well prepared to run a PC, although they still see the need to modify the curriculum to include more empirical experiences and administrative aspects related to conducting pharmaceutical services [25]. Likewise, interns from a Brazilian pharmacy school indicated that, despite assessing their pharmacy internships as satisfactory, they still saw the need to improve patient information, as well as interprofessional collaboration skills [26]. The presented research, similar to ours, shows that pharmacy students feel substantially prepared for the profession, while the education gap is still in the improvement of practical skills. Thus, a very important aspect that will enable the full implementation and development of pharmaceutical services in Poland is the satisfaction of pharmacy students with the curriculum, which may translate into the motivation to actively and creatively perform professional tasks in the future. Research highlights that the conviction of students to become equal members of the medical team taking care of the patient and their conviction concerning the high prestige of the pharmacy profession builds the confidence that their professional work will be satisfactory [27].

### 4.4. Students’ Attitudes towards the Applied Educational Solutions

The evaluation of the previous classes in the field of professional counselingshowed that students highly appreciate the knowledge of the lecturers, but that the teachers lacked more activating teaching methods. Moreover, students had a problem with defining the detailed learning outcomes which, according to the subject syllabus, relate to most aspects of running a PC. It can be assumed that the solution that would increase students’ awareness of the knowledge and skills required by a pharmacist running a PC is to increase the participation in classes by using active learning methods in the pharmacy curriculum, such as case-study, role-play, hands-on exercises, and simulation. Many studies around the world show that the inclusion of these kind of methods in the pharmacy program meets with a positive reaction from students, not only increasing the level of students’ knowledge, but above all, teaching the practical use of knowledge, critical thinking, decision making, and effective communication with the patient [28,29,30,31,32,33,34,35,36]. Students planning to work in a community pharmacy should be prepared and have the above mentioned competences to provide patient-centered services. Moreover, research shows that factors related to students’ inner beliefs and personality also influence the possibility of the effective use of knowledge in the provision of pharmaceutical services [37]. According to Ajzen’s concept of planned behavior, properly designed interventions can lead to the modification of internal beliefs, thus influencing behavior by directly influencing intentions [38]. The theory of planned behavior assumes that the factors influencing intentions are attitudes towards behavior, subjective norms and behavioral control. In terms of behavioral control, the author emphasized that, apart from actual behavioral control, an important and modifiable element is the subjective assessment of one’s own resources, i.e., the perception of behavioral control [38,39]. Although pharmacy students in Poland take many classes to prepare them substantively for the profession, they may present reduced self-efficacy. According to Bandura’s theory, self-efficacy is a belief in one’s own abilities to achieve a certain goal under various conditions, especially in stressful situations [40,41]. Whereas the sense of self-efficacy is related to internal beliefs and emotional well-being, it is possible to shape it, e.g., by gaining professional experience in controlled conditions [42]. Therefore, referring to the cited theory of planned behavior, low self-efficacy affects the perception of behavioral control, which in turn, influences the shaping of the intention to undertake specific behaviors, for example, in this case, providing a PC. Research in Malaysia has shown that the perception of behavior control has an impact on the self-esteem of pharmacy students regarding their knowledge of pharmacotherapy safety optimization [43]. Moreover, the experience throughout the world shows that the introduction of new educational solutions has a positive effect on the sense of self-efficacy of pharmacy students, although this is still an area that requires further research and new teaching concepts, especially in the field of a PC teaching [44,45,46,47,48]. For this reason, this study is part of a project aimed at the evaluation of educational solutions used thus far in the field of pharmacy, identifying students’ attitudes, introducing exercises using active teaching methods, and assessing their impact on the self-efficacy of students and their readiness to provide pharmaceutical services in accordance with Bandura’s theory.

## 5. Conclusions

Surveys conducted among students of the last year of pharmacy training at the PUMS revealed that they perceive pharmaceutical care as the obligation of a community pharmacist. They also emphasized that classes on a PC and internships allow them to improve the ability to use the acquired knowledge in pharmacy practice, which, as a consequence, increase their readiness to provide pharmaceutical services in their future work. Nonetheless, they pointed out that the direction for curriculum renewal should be the inclusion of more classes on pharmaceutical care using active learning methods.

The study allows for the identification of trends and relationships that can be the subject of in-depth research and contribute to the curriculum renewal at pharmaceutical institutions in Poland. Further research is needed to introduce and evaluate new educational solutions, such as simulation exercises, and to assess their impact on the readiness of future pharmacists to provide pharmaceutical care services in Poland.

## Figures and Tables

**Table 1:** Examples of learning outcomes related to the implementation of pharmaceutical care services, as defined in the Regulation of the Minister of Science and Higher Education of 26 July 2019.

Pharmaceutical Practice	
**Knowledge:** **Student knows and understands…**	the idea of pharmaceutical care and concepts related to pharmaceutical care, in particular relating to problems and needs related to the use of drugs.
	principles of monitoring the effectiveness and safety of patient pharmacotherapy in the process of pharmaceutical care.
	the role of pharmacists and representatives of other medical professions in a therapeutic team
	principles of health promotion, its tasks, and the role of a pharmacist in promoting a healthy lifestyle.
**Skills:** **Student can…**	plan, organize and conduct pharmaceutical care.
	conduct pharmaceutical consultations in the process of pharmaceutical care and pharmaceutical consulting.
	choose over-the-counter medications in diseases that do not require medical consultation.
	indicate the correct way to deal with the drug during its use by the patient and provide information about the drug.
	educate the patient about their medications and other problems related to their health and disease, and prepare personalized educational materials for the patient.

**Table 2:** Demographic structure of the respondents in the pharmaceutical care survey.

Sex (*n* = 64)	Median Age (Years) (*n* = 64)
Female *n* = 53	23.7
Male *n* = 11	24.6

**Table 3:** Students’ attitudes toward teaching and learning pharmaceutical care during the undergraduate pharmacy program.

Statement ^1^		Σn	n_1+2_	n_3_	n_4+5_	Mo	M	Q_1_	Q_3_
**Presence of pharmaceutical care in undergraduate curriculum**									
1	The PC classes have a positive effect on the professional preparation of students for the profession of pharmacist	64	5	2	57	5	5	4	5
2	PC should only be taught in elective courses and not in compulsory classes	64	60	1	3	1	1	1	1
3	There are too few hours of classes on PC in the curriculum	64	8	4	52	5	5	4	5
4	There are enough PC classes in the study program	64	51	5	8	1	2	1	2
5	PC classes should start in the fifth year of studies	63	54	3	6	2	2	1	2
6	Students should run a PC during their internship or apprenticeship	64	12	8	44	5	4	3	4.5
**Teaching content**									
7	PC classes should cover the practical aspects of using self-monitoring devices, e.g., glucometers, blood pressure monitors	64	2	0	62	5	5	5	5
8	PC classes should cover the practical aspects of using specific drug forms, e.g., pens, inhalers	64	2	0	62	5	5	5	5
9	PC classes should be about drug interactions	64	1	2	61	5	5	4	5
10	The PC class should focus on detecting drug problems	64	1	5	58	5	5	4.5	5
11	PC classes should teach the right approach to the patient	63	1	0	62	5	5	5	5
12	In PC classes, students should practice interviewing a patient	64	1	3	60	5	5	4	5
**Educational methods**									
13	PC classes should be based mainly on role-play	64	18	28	18	3	3	2	4
14	During PC classes, the tutor should initially present theoretical issues (e.g., in pharmacology) related to a given topic	64	13	9	42	4	4	3	4.5
15	The theory of a given topic should not be presented in PC classes	62	52	4	6	2	2	1	2
16	PC classes should consist only in practicing the use of previously acquired knowledge in practice	64	36	11	17	2	2	2	4
17	PC classes should be attended by external people (e.g., actors) professionally prepared to play the role of a patient	64	26	17	21	3	3	2	4
18	The scenes practiced by the participants should be recorded and then played back and discussed by the group and the teacher	64	37	11	16	2	2	2	3.5

Σn—total numberof respondents; n_1+2_—number of respondents who selected answers ‘strongly disagree’ or ‘disagree’; n_3_—number of respondents who selected answer ‘neutral’; n_4+5_—number of respondents who selected answers ‘agree’ or ‘strongly agree’; Mo—mode; M—median; Q_1_—lower quartile; Q3—upper quartile. ^1^ range of responses: 1—strongly disagree, 2—disagree, 3—neutral, 4—agree, 5—strongly agree.

**Table 4:** Students’ knowledge and attitudes toward pharmaceutical care.

Statement ^1^		Σn	n_1+2_	n_3_	n_4+5_	Mo	M	Q_1_	Q_3_
**Knowledge about pharmaceutical care service in Poland**									
1	Currently, it is impossible to run a PC in Polish pharmacies	63	15	14	34	4	4	2.5	4
2	In practice, pharmacists in Polish pharmacies already run a PC	64	40	16	8	2	2	2	3
3	A separate room in the pharmacy is required to run a PC	64	5	4	55	5	4	4	5
4	PC is mainly based on a patient’s history of medications	64	28	7	29	4	3	2	4
5	PC is mainly based on patient education	64	15	5	44	4	4	3	4
6	PC applies only to patients with chronic diseases	64	38	6	20	2	2	1.5	4
**The status of pharmaceutical care as a service provided by pharmacists**									
7	Running a PC has a real impact on improving the health of patients	64	2	1	61	5	5	4	5
8	I believe that running PC by pharmacists is entering into the competences of phycisians	64	61	2	1	1	1	1	2
9	Running a PC should be the responsibility of every pharmacist in a pharmacy	64	11	14	39	4	4	3	5
10	Pharmacists should not be required to run a PC	64	41	12	11	1	2	1	3
**Pharmacists’ readiness for providing pharmaceutical care**									
11	PC can only be handled by specialized pharmacists	64	5	12	47	4	4	3	5
12	I believe that pharmacists have too little knowledge to run a PC	64	37	13	14	2	2	2	3
13	I believe that studies prepare well for pharmacists to run a PC	63	29	16	18	2	3	2	4
14	In my opinion, the classes I have participated in so far have prepared me well to conduct education on the use of self-monitoring devices	64	9	10	45	4	3	2	4
15	In my opinion, the classes I have participated in so far have prepared me well to conduct education on the use of various forms of the drug, e.g., inhalers, pens	64	11	11	42	4	4	3	4

Σn—total number of respondents; n_1+2_—number of respondents who selected answers ‘strongly disagree’ or ‘disagree’; n_3_—number of respondents who selected answer ‘neutral’; n_4+5_—number of respondents who selected answers ‘agree’ or ‘strongly agree’; Mo—mode; M—median; Q_1_—lower quartile; Q_3_—upper quartile. ^1^ range of responses: 1—strongly disagree, 2—disagree, 3—neutral, 4—agree, 5—strongly agree.

**Table 5:** Demographic structure of the respondents in the evaluation survey.

Sex (*n* = 118)	Median Age (Years) (*n* = 118)
**Female *n* = 93**	23.9
**Male *n* = 25**	24.8

## Data Availability

The data presented in this study are available on request from the corresponding author. The data are not publicly available due to not having an appropriate repository to store questionnaires in digital form.
